# A European paramedic curriculum for geriatric emergency medicine developed via a modified Delphi technique

**DOI:** 10.1186/s13049-026-01550-3

**Published:** 2026-01-12

**Authors:** Jan-Niklas Krohn, Jack Barrett, Pieter Heeren, Stephen Lim, Elizabeth Moloney, Christian H. Nickel, James van Oppen, Nicolai Sandig, Luca Ünlü, Katrin Singler

**Affiliations:** 1 Department of Geriatrics, Klinikum Nürnberg, Paracelsus Private Medical University, Prof. Ernst-Nathan-Str. 1, 90419 Nuremberg, Germany; 2 South East Coast Ambulance Service NHS FT, Crawley, England, UK; 3 Faculty of Medicine and Life Sciences, Hasselt University, 3590 Agoralaan, Diepenbeek Belgium; 4 Department of Public Health and Primary Care, Academic Centre for Nursing and Midwifery, KU Louvain, Kapucijnenvoer 7 bus 7001, 3000 Louvain, Belgium; 5 National Institute for Health and Care Research Applied Research Collaboration Wessex, Academic Geriatric Medicine, University of Southampton, Southampton, UK; 6 Department of Geriatric Medicine, Mercy University Hospital, Cork City, T12 WE28 Ireland; 7 Department of Emergency Medicine, University Hospital Basel, University of Basel, Petersgraben 2, 4031 Basel, Switzerland; 8 Centre for Urgent and Emergency Care, University of Sheffield, Sheffield, S1 4DA UK; 9 AGNF, Institute for Emergency Medical Education, Deutenbacher Str. 1, 90547 Stein, Germany; 10 Department of Emergency Medicine, Medical Center-University of Freiburg, Medical Faculty-University of Freiburg, Freiburg, Germany; 11 Department of Geriatrics, Klinikum Fürth, Fürth, Germany; 12 Institute for Biomedicine of Ageing, Friedrich-Alexander University Erlangen-Nürnberg, Nuremberg, Germany

**Keywords:** Prehospital care, Emergency medicine, Geriatrics, Older adults, Paramedic education, Curriculum development, Delphi technique, Learning objective, Competence-based education, Europe

## Abstract

**Background:**

Older emergency patients currently account for most European emergency medical service dispatches. Due to demographic changes and increasing comorbidities in advanced age, this number is expected to rise substantially in the coming years. Prehospital professionals require specialised training to provide high-quality care for complex, multimorbid patients. The aim of this study is to define minimum competencies for paramedic education in Europe on the management of emergencies in older adults.

**Methods:**

A modified electronic Delphi study was performed from January 2023 to November 2024, comprising two appraisal rounds. A narrative literature review was conducted to identify relevant topics and domains in prehospital geriatric emergency medicine, providing the foundation for an interprofessional core group to establish 58 initial learning objectives. Learning objectives were assigned to competence levels based on a revised Bloom's Taxonomy.

**Results:**

In Round 1, 45 of 58 competence-based learning objectives were accepted (77.6%) with average agreement 83.2% [range: 70.8–93.9%]. 13 declined learning objectives were revised, including merging and splitting of learning objectives, adjusting competence levels, and grouping domains. In Round 2, all 12 adapted learning objectives were accepted with average agreement 87.1% [range: 75–100%]. The final curriculum has 57 learning objectives in 12 domains. This consensus was achieved with contributions from Delphi panellists across 27 European countries. The domains include: risk stratification; indicators of serious health problems; altered mental status; clinical assessment; falls; trauma; medication; communication; medical history; frailty; palliative and end-of-life care; positioning and transport; and social, psychological and legal aspects.

**Conclusions:**

This European curriculum for prehospital geriatric emergency medicine represents a first step towards systematically integrating these geriatric-specific competencies into paramedic education. It can further serve as a foundation for standardised training programs aimed at addressing the complex needs of older emergency patients.

**Supplementary Information:**

The online version contains supplementary material available at 10.1186/s13049-026-01550-3.

## Background

### Older patients in emergency medical services

Emergency medical services (EMS) were initially designed to treat life-threatening conditions [1], commonly employing algorithms and protocols to guide rapid treatment and transport decisions. This contrasts with the holistic, person-centred approach to decision-making and treatment from which older people living with frailty are known to benefit [2]. However, as populations have grown and aged, there has been a shift in demand for EMS, which are required to provide unscheduled urgent and emergency care. Today, most patients transported by ambulance are older adults (65 years and above) [3–6]. Older EMS users have significantly higher prevalence of frailty [7–9] compared with the general population [10, 11]. Caring for these patients can be challenging because they are a heterogeneous population often with complex medical, psychological and social conditions [12]. Appropriate recognition of the patient's needs by EMS has profound implications for patient experience and health outcomes [13]. Concerningly, prehospital diagnostic accuracy decreases from the age of 60 years, as shown by retrospective comparisons of prehospital diagnoses with the corresponding intrahospital medical records [14]. Furthermore, 1-day and 30-day mortality after EMS call increases with advanced age [15]. For these reasons, geriatric emergency care is of increasing importance across European countries [3, 16, 17].

### *Importance*

European research on paramedic education in geriatric emergency medicine is limited, and at the same time paramedic training curricula and scope of practice varies from country by country [18]. Previous surveys among paramedic students describe an educational gap in geriatric medicine [19, 20]. Despite their generally positive attitudes towards older adults, they showed poor factual knowledge on ageing [19]. It has been shown that a two-hour workshop followed by patient-centred interviews with older people improves the communication skills of paramedic students, particularly by enhancing their understanding of the patient’s perspective [20]. Moreover, an American interventional study demonstrated that a 1-day Geriatric Emergency Medical Service (GEMS) course positively impacts EMS providers’ comfort in communicating with older adults, caring for their medical conditions, performing fall risk assessments, and assessing abuse or neglect [21]. While the National Association of Emergency Medical Technicians is addressing the underrepresentation of geriatric medicine in undergraduate paramedic training with the GEMS course, to date no consensus-derived paramedic curriculum for geriatric emergency medicine has been proposed.

### Aim of this study

This study aimed to develop a widely accepted expert recommendation for minimum geriatric emergency medicine competencies in European paramedic education. For this study, paramedics are defined as individuals with the highest level of prehospital training, routinely practicing in EMS in their countries. Excluded are physicians, individuals with only basic or non-standardised EMS training, and those with higher academic degrees, who are additions rather than standard staffing on ambulances in their respective countries. Educational backgrounds may include various levels of post-secondary education, such as vocational training programs, dual training, or undergraduate programs. A modified Delphi method was used to systematically achieve expert consensus on essential competencies, ensuring that educational content reflects both current evidence and professional standards.

## Methods

**Fig. 1:**
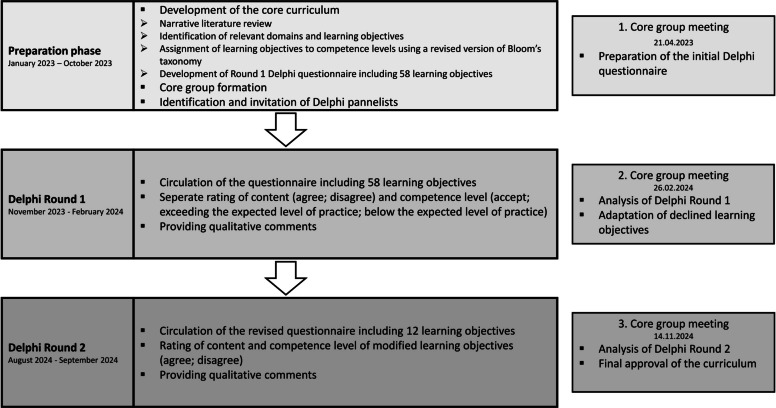
Graphic illustration of the Delphi process

This modified electronic Delphi study was undertaken from January 2023 to November 2024 and included two Delphi rounds (Fig. 1). Reporting is performed in accordance with the ACCORD protocol [22]. The study was prospectively registered at the Research Management and Service of Klinikum Nuremberg (trial registration number: FMS_W_099.22-II-2) in July 2022. This project was exempted from ethics committee approval under the Medical Research Act. All participants provided informed consent and participated voluntarily.

### Core group

Core group formation was iterative, with new members invited as needs were identified by the founders (KS and JNK) and existing committee. Potential members were identified via literature research, professional connections, and network recommendations. The final core group included international healthcare professionals specialised in prehospital care, geriatric medicine and nursing, emergency medicine, and methodologists who provided expertise in the execution of the Delphi process. Educationalists experienced in curriculum development and paramedic education were also included in the expert group. The core group's role was to facilitate two rounds of Delphi Study voting among a broader membership. Specifically, core members met on three occasions online and contributed to the preparation of initial statements from literature review evidence (21.04.2023), analysis and review of Delphi Rounds as well as adaption of the learning objectives (26.02.2024), and approval of the final curriculum (14.11.2024) (Fig. 1).

### Development of the core curriculum

As there are no existing consensus-derived paramedic curricula for geriatric emergency medicine, the core curriculum development was guided through a narrative literature review aimed at identifying relevant topics and domains in geriatric emergency medicine within prehospital emergency care, complemented by discussions within the core group that reinforced the importance of focusing on key competencies for the prehospital care of older adults. Potentially relevant papers were identified from 1 database (PubMed) and 1 search engine (Google Scholar) using the following search terms in appropriate strings: older patients, geriatric, paramedics, Emergency Medical Services, emergency medicine, education, training. The search was supplemented by grey literature, personal collections and by manual screening of the references in selected papers.

Based on the literature review findings, the core group generated a list of domains and a range of items for potential inclusion in the new curriculum. These were discussed openly and modified following the principle of consensus.

The existing European Geriatric Emergency Medicine curriculum [23], designed for the emergency department setting, was examined for domains and items potentially relevant to the prehospital setting and furthermore served as the basis for the development of competence-based learning objectives. The competence-based learning objectives, defined as specific, measurable statements describing the knowledge, skills or behaviours paramedics are expected to demonstrate at the end of their training, were derived from the content of the identified papers rather than explicitly stated within them. Furthermore, learning objectives were assigned to competence levels based on a revised version of Bloom’s taxonomy [24]. For this purpose, a separate Bloom’s taxonomy framework was developed, guiding the evaluation of paramedic students’ competencies in geriatric emergency care (Supplementary file 1).

### Delphi panel

European experts in the field of emergency medicine, geriatric emergency medicine as well as experts on paramedic education were invited to participate in February 2023. Potential participants for the Delphi panel were identified via professional connections of KS and JNK, the European Taskforce on Geriatric Emergency Medicine (www.geriemeurope.eu) and other network recommendations as well as professional connections of other core group members via e-mail. Invited panellists were asked to suggest additional experts, through which further participants were identified. Moreover, in countries where no panellists could initially be identified, national professional societies and EMS organisations were contacted to identify further potential panellists. A total of 88 survey links were sent to individuals who had confirmed their participation. No direct incentives were offered; however, participants were acknowledged in the final publication. To encourage participation, invitations and reminder emails were sent repeatedly. None of the core group members participated in the Delphi panel. All participants were asked to provide personal information and information about the EMS as well as paramedic education in their respective countries. Prior to Round 1, all participants received the revised Bloom's taxonomy (Supplementary file 1) and information on the objectives of the new curriculum.

### Consensus

Two Delphi rounds were undertaken. These were conducted remotely and anonymously. Panellists could provide general feedback as well as individual comments on the content and competence level of each learning objective. The online questionnaires were sequentially piloted by two independent individuals to ensure system performance and clarity of the items.

Acceptance rate of each learning objective and its respective competence level was defined a priori. Acceptance thresholds were guided by the European postgraduate curriculum in geriatric medicine [25] and defined as follows: learning objectives with over 70% agreement were accepted, those with 50–70% agreement were reviewed in a second round and those with less than 50% agreement were predetermined to be rejected. The core group decided to exclude all responses, if less than 50% of the learning objectives were reviewed in the second core group meeting to ensure data quality and to avoid bias from incomplete questionnaires.

#### Delphi Round 1

In Round 1, the Delphi panellists were asked to rate the content of the learning objectives using a dichotomous scale (agree/disagree). Competence level of the respective learning objective was asked only after accepting the content (accept; too high = exceeding the expected level of practice; too low = below the expected level of practice) to minimise survey completion time. Disagreement with content or competence level was summarised to determine the agreement rate of the complete learning objective.

#### Delphi Round 2

Before Round 2, formal feedback was provided, including a summary of Delphi Round 1 and a rationale for the modifications to the rejected learning objectives based on panellists’ ratings and comments. In Round 2, Delphi panellists were asked to rate the complete learning objectives, encompassing both content and competence level, using a dichotomous scale (agree/disagree).

## Results

### Delphi panal characteristics

In Round 1, 56 experts participated. After excluding seven submissions with fewer than 50% of learning objectives reviewed, 49 participations were included in the analysis (87.5%). In Round 2, 43 experts participated. After excluding three submissions with fewer than 50% of learning objectives reviewed, 40 participations were included in the analysis (93%).

**Fig. 2:**
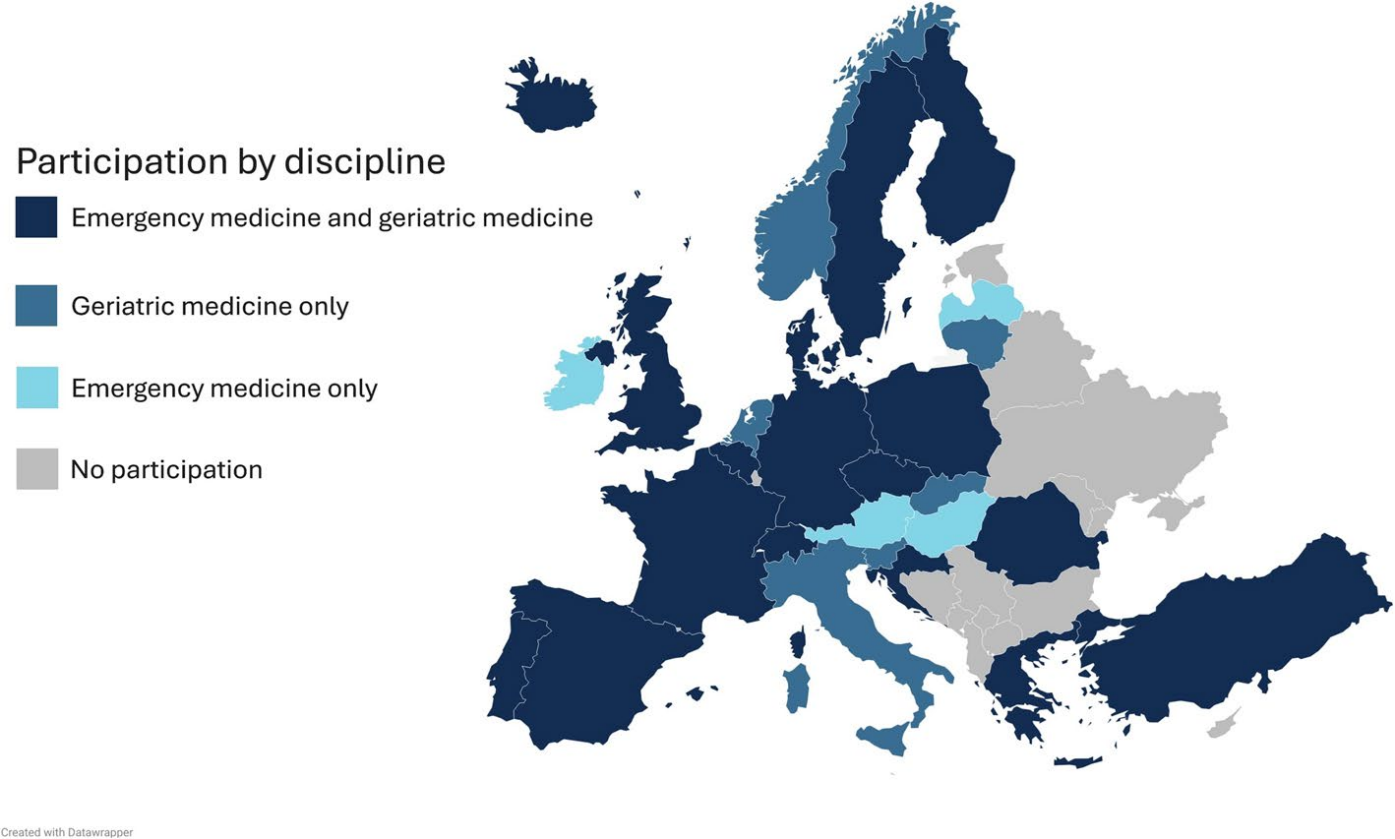
European distribution of Delphi panel members’ nationalities by specialty. Experts from 27 European countries participated in the Delphi process. 17 countries were represented by geriatric and emergency medicine representatives. Six countries were represented only by geriatric representatives, and four were represented only by emergency medicine representatives

In total, experts from 27 European countries participated in the Delphi process (Fig. 2). As some experts had expertise in both, geriatrics and emergency medicine, all panellists were asked to identify with one of the disciplines (Table 1, Supplementary file 2). Most countries were represented by both geriatric and emergency medicine representatives [17]. Six countries were represented only by geriatric representatives, and four were represented only by emergency medicine representatives (Fig. 2). Further socio-demographic data of the Delphi panellists is provided in Table 1 and Supplementary file 2.

**Table 1:** Description of the expert panel during the Delphi process

Data	First round (%)	Second round (%)
**Participations**		
Total inclusions	49 (100)	40 (100)
**Main field of expertise**		
Geriatric medicine	25 (51)	25 (62.5)
Emergency medicine	24 (49)	15 (37.5)
**Years of experience in main field of expertise**		
More than 10 years	30 (61.2)	25 (62.5)
5 to 10 years	15 (30.6)	10 (25)
Less than 5 years	4 (8.2)	5 (12.5)
**Profession** (multiple answers possible)		
Medical doctor	37 (75.5)	35 (87.5)
Paramedic	6 (12.2)	4 (10)
Emergency Medical Technician	3 (6.1)	0
Nurse	2 (4.1)	2 (5)
Educator/Instructor/Teacher	10 (20.4)	9 (22.5)
Other	5 (10.2)	2 (5)

### Paramedic education and EMS structure

The duration of paramedic training in Europe varies between less than one year and more than four years (Table 2). Training is provided predominantly at universities and vocational schools, with some programs offered by specialised paramedic training institutions. Responses from the Delphi panellists suggested that paramedics from different countries would apply different strategies for emergency patient care, most commonly selecting a suitable strategy as described in Table 2, depending on the scenario.

**Table 2:** Expert panel perceptions of paramedic education and strategies in prehospital care

Data	First round (%)	Second round (%)
Participants	49 (100)	40 (100)
**Educational institutions of paramedic training**		
University/College	20 (40.8)	15 (37.5)
Vocational School	15 (30.6)	12 (30)
Other	9 (18.4)	8 (20)
I do not know	5 (10.2)	5 (12.5)
**Duration of paramedic training**		
Less than 1 year	8 (16.3)	5 (12.5)
1 year	5 (10.2)	6 (15)
2 years	6 (12.2)	3 (7.5)
3 years	16 (32.7)	16 (40)
4 years	9 (18.4)	3 (7.5)
More than 4 years	1 (2)	2 (5)
I do not know	4 (8.2)	5 (12.5)
**Predominant strategies for delivering care**		
Load and Go: Quickly transporting the patient to the hospital	3 (6.1)	3 (7.5)
Treat and Run: Providing initial treatment on scene, then transporting	12 (24.5)	10 (25)
Stay and Play: Providing extended on-scene care before transport	1 (2)	2 (5)
EMS personnel choose the most suitable strategy depending on the scenario	29 (59.2)	20 (50)
Other	0	1 (2.5)
I do not know	4 (8.2)	4 (10)

#### Round 1

Round 1 took place from November 2023 to February 2024 and involved the expert panel voting on 58 draft learning objectives. In total, 45 of the 58 learning objectives were accepted (77.6%) with an average agreement of 83.2% [range: 70.8–93.9%], including the expected competence level. The acceptance rate of the content of the learning objectives was 98.3% (n = 57), with an average agreement of 91.2% [range: 73.5–98%].

In total, thirteen learning objectives were declined with an average agreement of 62.8% [range: 51 – 69.4%] (Supplementary file 3). For one learning objective, the content was declined, while the remaining twelve were declined considering both content and competence level (Supplementary file 3). For the 13 declined learning objectives, the assigned competence level was considered too high in 66.4% [range: 43–92%] of cases, and too low in 33.6% [range: 8–57%], respectively.

Consensus was reached for six domains: *indicators of serious health problems, trauma, medication, communication and medical history taking, frailty, positioning and transport, and social, psychological, and legal aspects* (Supplementary file 3).

### Adaptation of declined learning objectives

The core group adapted all 13 rejected learning objectives, considering expert panellists’ comments. This involved merging two pairs of objectives, separating one learning objective, and removing one domain. This resulted in 12 revised objectives (Supplementary file 3).

The learning objective regarding *“diminished decision-making capacity and care that preserves autonomy and self-determination”* was separated into two individual learning objectives to recognise both topics accordingly. The two learning objectives regarding *“medication-based pain management”* and *“medication in geriatric patients”* were merged due to the significant overlap of learning objectives, which emerged after Round 1 results. The domain regarding pain was removed, enhancing overall clarity. The remaining learning objective addressing *“identification of pain”* was transferred to the domain *“clinical assessment”*. Furthermore, two learning objectives relating to *“legal and regulatory frameworks surrounding end-of-life care”* as well as *“advance directives and powers of attorney”* were merged due to the significant overlaps of learning objectives. This emerged after Round 1 feedback highlighting the lack of legal regulations in some European countries. Other modifications were made to adapt the competence levels of certain learning objectives (Supplementary file 3) to align with the expert panel’s rating of the expected level of practice, minimising the number of included examples and improve overall clarity.

#### Round 2

Round 2 took place from August 2024 to the end of September 2024. All 12 adapted learning objectives were accepted with an average agreement of 87.1% [range: 75–100%] (Supplementary file 3). The core group approved the final curriculum consisting of 57 learning objectives within 12 domains (Table 3) in November 2024. In the final curriculum, most learning objectives were assigned to the competence levels *Understand* (*n* = 19; 33.3%) and *Analyse* (*n* = 16; 28.1%), followed by *Apply* (*n* = 12; 21.1%) and *Remember* (*n* = 10; 17.5%) within the cognitive process dimension. Regarding the knowledge dimension, the majority were classified as *Conceptual knowledge* (*n* = 33; 57.9%), with fewer assigned to *Procedural knowledge* (*n* = 14; 24.6%), *Metacognitive knowledge* (*n* = 8; 14%), and *Factual knowledge* (*n* = 2; 3.5%) (Table 3).

**Table 3:** European paramedic curriculum in geriatric emergency medicine

Domain 1: Risk stratification	
1	To be able to identify high risk situations independent of algorithm-based workflows
2	To be able to consider frailty as a parameter for risk stratification
3	To be able to recognise patients’ physical and cognitive ability and resilience towards acute medical interventions
4	To be able to correctly interpret vital signs (including respiratory rate, SpO2, pulse, blood pressure, blood sugar, mental status and pain)
5	To be able to demonstrate awareness of the limitations of conventional physiological parameters in identifying older patients with serious acute illness
6	To be able to identify atypical symptoms of serious acute diseases
Domain 2: Indicators of serious health problems	
7	To be able to identify falls as a potential indicator of possible serious underlying health problems
8	To be able to identify a recent decline in activities of daily living as an indicator of possible serious underlying health problems
9	To be able to identify generalised weakness as an indicator of possible serious underlying health problems
10	To be able to identify altered mental status as a potential indicator of potential serious acute illness
Domain 3: Altered mental status	
11	To be able to identify an altered mental status in older emergency patients
12	To be able to differentiate between acute and chronic alterations in mental status
13	To be able to consider common causes of acute altered mental status (e.g. pain, hypoxia, hypoglycaemia, trauma) and to initiate emergency management
14	To be able to identify delirium as an emergency
15	To be able to recall the predisposing and precipitating factors of delirium
16	To be able to recognise diminished decision-making capacity
17	To be able to deliver care that respects and preserves autonomy and self-determination
Domain 4: Clinical assessment	
18	To be able to describe the impact of underlying cognitive disorders on the clinical assessment
19	To be able to recognise clinical signs of severe infection and sepsis and to deliver initial management (e.g. intravenous fluids)
20	To be able to recognise clinical signs of cardiovascular emergencies and to deliver individualised management
21	To be able to recognise clinical signs of neurological emergencies and to deliver individualised management
22	To be able to recognise clinical signs of dehydration and malnutrition and to deliver individualised management
23	To be able to recognise clinical signs of mental health crises (including depression and suicidal behaviour) and to initiate interventions (including referral to specialists)
24	To be able to recognise clinical signs of abuse (including physical, psychological, social abuse) and to deliver individualised management
25	To be able to recognise the impact of underlying gait and movement disorders (e.g. Parkinson’s disease) on clinical assessment
26	To be able to identify pain in patients with and without cognitive impairment (including the use of standardised assessment tools)
Domain 5: Falls	
27	To be able to perform a basic falls assessment, including history-taking, physical examination, and functional assessment
28	To be able to consider influencing factors on transport decisions such as severity of injuries, clinical condition, risks and benefits of transport, patient preferences and potential underlying causes
Domain 6: Trauma	
29	To be able to conduct a systematic and focused evaluation of older trauma patients, considering both obvious and subtle injuries whilst assessing the severity of the trauma
30	To be able to perform individualised management for patients with low energy transfer trauma
Domain 7: Medication	
31	To be able to obtain a medication history (including over-the-counter products, frequency, compliance, recent changes) and to produce a structured report
32	To be able to appreciate the importance of a detailed medication list for subsequent healthcare
33	To be able to identify high risk medication (e.g. anticoagulants, anti-platelets, anti-diabetics, antiarrhythmic drugs, diuretics, cholinergic drugs)
34	To be able to consider adverse drug events as possible cause of clinical presentation
35	When administering drugs: To be able to adapt medication (dosage) taking into consideration age-related physiological changes and comorbidities
Domain 8: Communication and medical history taking	
36	To be able to explain the impact of person-centred communication
37	To be able to describe the role of professional communication with patients, relatives and health care providers
38	To be able to adapt communication skills to the individual patient needs to support shared decision-making
39	To be able to perform a focused medical history taking in patients with cognitive and functional impairments
40	To be able to perform a structured collateral history including social care
41	To be able to optimise preexisting sensory deficits (e.g. the use sensory aids such as visual and hearing aids) to overcome communication barriers
Domain 9: Frailty	
42	To be able to describe the concept of frailty and its implications
43	To be able to appreciate the risks and benefits of attending the emergency department for frail persons
44	To be able to identify frailty in older emergency patients
Domain 10: Palliative and End-of-life care	
45	To be able to consider patient-centred healthcare goals based on the patient’s condition, perspectives, and the potential benefits and risks of various interventions (e.g. hospitalisation)
46	To be able to recognise palliative care needs and to initiate physical, psychological, and social support
47	To be able to explain the importance of effective communication and compassionate support when delivering end-of-life care to patients and their families
48	To be able to recall the relevant legal and regulatory frameworks surrounding palliative care decisions, where available
49	To be able to analyse personal attitudes, biases, and emotions related to death and dying, and develop strategies for self-care and professional resilience when providing end-of-life care
Domain 11: Positioning and transport	
50	To be able to consider patient-specific factors (e.g. skin problems, pain) when determining the transportation route and positioning within the ambulance
51	To be able to optimise preexisting mobility deficits (e.g. to convey patients with their mobility aids) in order to overcome barriers to mobilisation
Domain 12: Social, psychological and legal aspects	
52	To be able to explain negative stereotypes associated with older people
53	To be able to analyse reasons for suboptimal care, encouraging self-reflection and promoting empathy towards older individuals
54	To be able to consider the importance of different community health care/social facilities for the care of older people
55	To be able to initiate support for relatives requiring assistance
56	To be able to appreciate patients’ preferences when delivering emergency care
57	To be able to integrate healthcare proxy holders (e.g. family, friends, or caregivers) when necessary

## Discussion

This modified Delphi study established 57 learning objectives across 12 domains, defining the minimum competencies for paramedic education in Europe regarding the prehospital management of emergencies in older adults.

The work of paramedics is continuously evolving in response to demographic development and changes in health policy. In modern EMS systems, the care of older patients has become a critical component of paramedic practice, largely due to the high call volume involving this population [3, 5] and the significantly higher mortality rate, with a 30-day mortality of 9% in patients above 60 years compared to 2% in those aged 31–60 [15]. This is the first paper to address the issue of paramedic training in geriatric emergency medicine at a European level.

Based on findings from our literature review, core group discussions and the Delphi process, the 12 domains and 57 competence-based learning objectives of this curriculum (Table 3) comprehensively address the challenges EMS face in the unscheduled care of older patients. In addition to patient benefit, paramedics themselves derive significant advantages from geriatric medicine-attuned training, as it enhances both their confidence and competence when caring for older adults [21] and their communication skills, particularly in understanding patients’ wishes and perspectives [20].

Despite the diverse structure of the European EMS [26] as well as educational differences across Europe [18, 27], defining a consensus on paramedic training in geriatric emergency care was feasible with high acceptance rates (Supplementary file 3). These findings demonstrate that the core challenges in prehospital geriatric emergency medicine directly shape the essential training requirements for paramedics and that these needs are not confined to individual countries. The expert group stated that paramedics apply different strategies for emergency patient care, most commonly selecting a suitable strategy depending on the scenario (Table 2). Although this flexibility promotes optimal patient outcomes, it consistently requires a high degree of professional expertise, which can be ensured only through appropriate training.

This curriculum complements existing European curricula in geriatric medicine, which define the minimal requirements that a medical student should achieve by the end of medical school [28], or that geriatricians [23, 25] and emergency physicians [23] should be able to demonstrate at the end of their specialty training. It also addresses geriatric syndromes such as frailty, delirium, multimorbidity, and falls [23, 25, 28]. Furthermore, this curriculum aligns with previous frameworks in competencies covering communication, clinical assessment and palliative care [23, 25, 28]. Serving as one of the guiding frameworks, the European Geriatric Emergency Medicine curriculum [23] defines 12 competencies in prehospital care. Beyond these, the new paramedic curriculum incorporates further learning objectives that are consistent with its principles. However, with a strong focus on applicability to prehospital care, several of the 98 competencies (e.g. interpretation of laboratory data) were not incorporated. Distinct from existing frameworks, the European paramedic curriculum for geriatric emergency care defines learning objectives that are uniquely relevant to paramedic care. For example, it addresses patient-specific factors when determining the transportation route and positioning within the ambulance. Furthermore, it is the first curriculum to consider influencing factors on transport decisions following a fall, including severity of injuries, clinical condition, patient preferences, potential underlying causes as well as risks and benefits of transportation.

### Strengths and limitations

This curriculum not only defines the content of paramedics’ geriatric education but also defines the competence level of each learning objective. As each learning objective is aligned with appropriate "action verbs" in the context of Bloom's taxonomy, the expected level of competence is precisely defined [29]. This provides a widely accepted framework for setting measurable competence levels, ensuring that paramedic students across different regions and institutions attain comparable standards. It is important to note that this curriculum was developed to complement competence-based training, which ensures that paramedics are able to prioritise and master decision-making in time-critical situations.

The learning objectives of this new curriculum include predominantly *Conceptual knowledge* (57.9%) followed by *Procedural knowledge* (24.6%) *and Metacognitive knowledge* (14%), with only 3.5% classified as *Factual knowledge*. This distribution addresses the complex medical, psychological and social conditions of older adults [12]. It underscores the importance of a patient-centred approach, which cannot be adequately managed through algorithm-driven approaches alone. Furthermore, integrating advanced levels of Bloom's taxonomy (28.1% *Analyse*, 21.1% *Apply*) allows for education that promotes critical thinking and decision-making.

Despite the robustness of the curriculum and the Delphi methodology, there are several limitations to consider. Participation in the Delphi panel was limited to English-speaking experts, as no translations were provided. Patient or public representatives were not included, leaving their perspectives unexplored. Furthermore, several experts withdrew from the Delphi process, most likely because of the long duration of the individual Delphi rounds and the overall length of the process. Although participants from 27 European countries were involved, the expert panel did not include representatives from all European countries, notably Eastern Europe (Fig. 2, Supplementary file 2), which may limit the generalisability of the curriculum to regions that were not represented.

It is important to note that panellist comments in Round 1 indicated that many considered the competence level assigned to the declined learning objectives to exceed the expected level of practice. Unfortunately, because the content had been rejected, the competence level was not assessed separately. Therefore, it is possible that the proportion of learning objectives for which the competence level was deemed too high (66.4%) was underestimated. For this reason, the adaptations made by the core group did not include any increases in the expected competence level. Furthermore, a separate assessment of content and competence level was not performed in Delphi Round 2.

Since there are different levels of training for EMS personnel in some European countries, this curriculum can be seen as a broader framework that spans multiple levels of training. As this curriculum is designed for training of paramedics, it may not be suitably comprehensive for those paramedics with extended scope or advanced practice. Consequently, certain aspects may be less relevant for those at the entry level. Additionally, some aspects of the curriculum may be less relevant for paramedics working in other clinical care settings such as primary care. Moreover, although we established minimum competencies for geriatric emergency medicine of paramedics in Europe, we did not establish a hierarchy among them. As a result, educational institutions may select contextually which learning objectives to adopt if full integration is not undertaken.

The disproportionately large number of medical doctors in the Delphi panel presents a further limitation, compounded by the limited data available on paramedic education in geriatric emergency medicine. This implies that further revisions may be necessary as the authors receive feedback from paramedic training bodies, paramedics students and paramedics after the curriculum's implementation in real-world practice. Future curricula should be developed in closer collaboration with EMS organisations and educational institutions to incorporate additional EMS-specific learning objectives, such as resources and risk factors in the patient's home environment and to establish a hierarchy among the learning objectives.

While the learning objectives were developed to be broadly applicable, certain topics (such as legal and regulatory frameworks) will require country-specific adaptation to ensure effective implementation. This need for contextual adaptation highlights the importance of collaboration with local stakeholders in each country to refine and embed these objectives within their specific EMS systems. Additionally, paramedic competencies and priorities differ significantly from those in the emergency department, where more research is available. As such, future curricula should integrate findings from prehospital care research to ensure that paramedic education is aligned with the unique demands of paramedic practice. In particular, further research is needed to determine the required level of comprehensiveness for specific geriatric interventions and how they can be effectively integrated into paramedic practice, ensuring they provide sufficient benefit. This is essential to justify the additional time investment, considering the operational responsibilities, including system preparedness, that paramedics are obligated to meet. Furthermore, research is needed to assess the feasibility of implementation of these learning objectives in current training programmes and the subsequent impact on patient outcomes.

## Conclusions

This first consensus-derived curriculum allows institutions and health systems to align their educational programmes in geriatric emergency medicine with a minimum standard recommended by an international expert panel.

## Supplementary Information

Supplementary Material 1. Revised Bloom’s taxonomy for the European paramedic curriculum for geriatric emergency medicine.

Supplementary Material 2. Detailed information on the expert panels’ composition.

Supplementary Material 3. Detailed information on acceptance rates and adaptation of learning objectives during the Delphi process.

## Data Availability

All data generated or analysed during this study are included in this published article and its supplementary information files.

## Abbreviation

EMS: Emergency medical service
