# Supplementary figures and images for: A European paramedic curriculum for geriatric emergency medicine developed via a modified Delphi technique
Source: Scand J Trauma Resusc Emerg Med. 2026 Jan 12;34:14. doi: 10.1186/s13049-026-01550-3 (PMC12849588; doi:10.1186/s13049-026-01550-3)

Knowledge Dimension

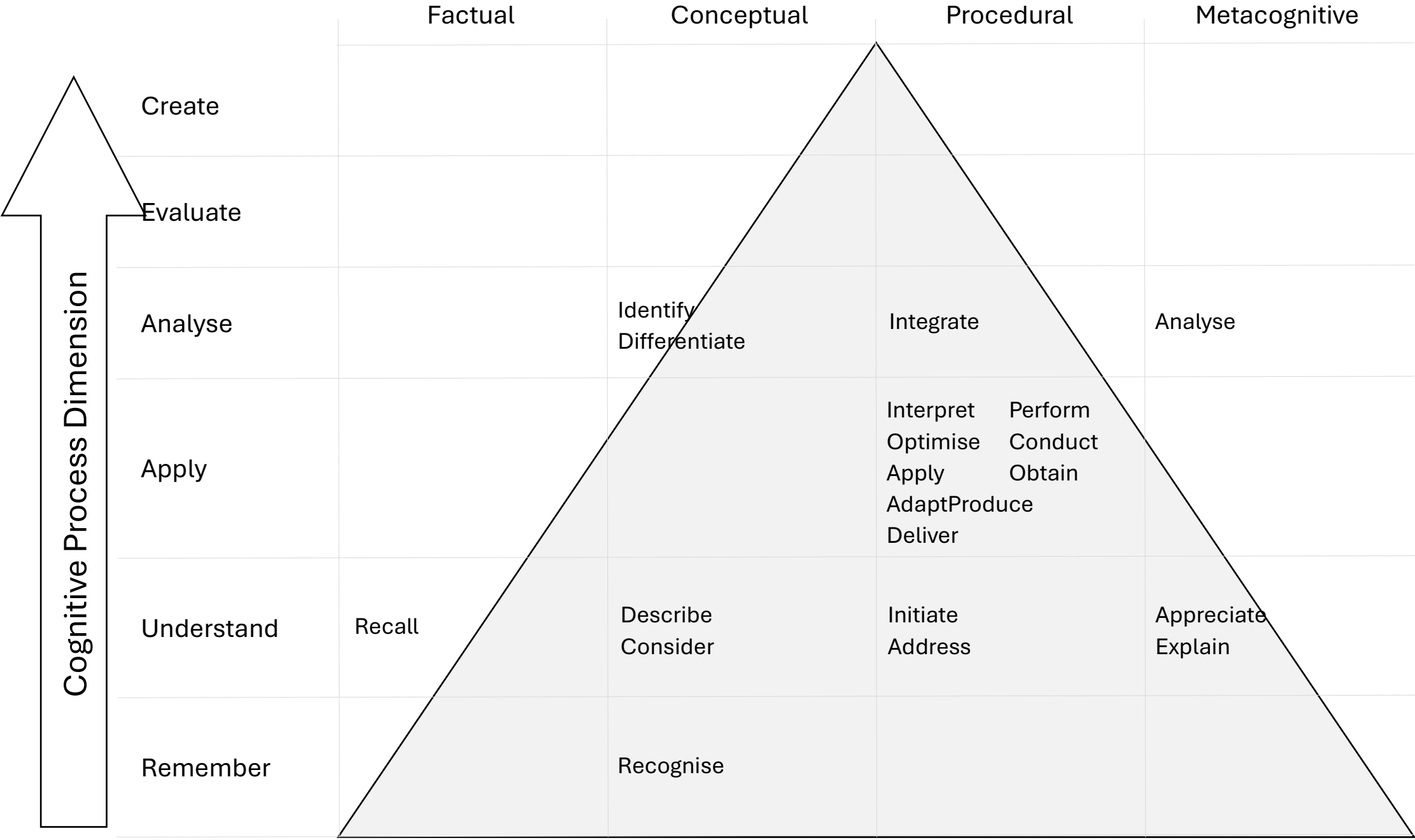

Supplement: Supplementary file 1 — Supplementary Material 1. Revised Bloom’s taxonomy for the European paramedic curriculum for geriatric emergency medicine. [file 13049_2026_1550_MOESM1_ESM.pdf]
