# Supplementary material for: A European paramedic curriculum for geriatric emergency medicine developed via a modified Delphi technique
Source: Scand J Trauma Resusc Emerg Med. 2026 Jan 12;34:14. doi: 10.1186/s13049-026-01550-3 (PMC12849588; doi:10.1186/s13049-026-01550-3)
Supplement: Supplementary file 2 — Supplementary Material 2. Detailed information on the expert panels’ composition. [file 13049_2026_1550_MOESM2_ESM.pdf]

Supplementary file 2. Detailed information on the expert panels' composition

Data	First round (%)	Second round (%)
**Participations**		
Total inclusions	49 (100)	40 (100)
**Gender**		
Male	26 (53,1)	22 (55)
Female	23 (46,9)	18 (45)
**Main field of expertise**		
Geriatric medicine	25 (51)	25 (62,5)
Emergency medicine	24 (49)	15 (37,5)
**Years of experience in the main field of expertise**		
More than 10 years	30 (61,2)	25 (62,5)
5 to 10 years	15 (30,6)	10 (25)
Less than 5 years	4 (8,2)	5 (12,5)
**Profession** (multiple answers possible)		
Medical doctor	37 (75,5)	35 (87,5)
Paramedic	6 (12,2)	4 (10)
Emergency Medical Technician	3 (6,1)	0
Nurse	2 (4,1)	2 (5)
Educator / Instructor / Teacher	10 (20,4)	9 (22,5)
Other	5 (10,2)	2 (5)
**Number of European countries represented**		
Total	25 (100)	24 (100)
Represented by 1 expert	8 (32)	11 (45,8)
Represented by 2 experts	11 (44)	11 (45,8)
Represented by 3 experts	5 (20)	1 (4,2)
Represented by 4 experts	1 (4)	1 (4,2)
**Number of included participations from respective country**		
Albania	-	-
Austria	3	2
Belgium	2	4
Bulgaria	-	-

Croatia	2	3
Czech Republic	2	2
Cyprus	-	-
Denmark	4	2
Estonia	-	-
Finland	2	1
France	1	2
Germany	3	2
Greece	2	0
Hungary	1	0
Iceland	3	2
Ireland	1	1
Italy	1	2
Latvia	1	0
Lithuania	1	1
Luxembourg	-	-
Malta	-	-
Moldavia	-	-
Montenegro	-	-
Netherlands	1	1
North Macedonia	-	-
Norway	0	1
Poland	2	2
Portugal	3	1
Romania	3	2
Serbia	-	-
Slovakia	0	1
Slovenia	1	1
Spain	2	1
Sweden	2	1
Switzerland	2	2
Turkey	2	2
United Kingdom	2	1
