# Supplementary material for: A European paramedic curriculum for geriatric emergency medicine developed via a modified Delphi technique
Source: Scand J Trauma Resusc Emerg Med. 2026 Jan 12;34:14. doi: 10.1186/s13049-026-01550-3 (PMC12849588; doi:10.1186/s13049-026-01550-3)
Supplement: Supplementary file 3 — Supplementary Material 3. Detailed information on acceptance rates and adaptation of learning objectives during the Delphi process. [file 13049_2026_1550_MOESM3_ESM.pdf]

Supplementary file 3: Detailed information on acceptance rates and adaptation of learning objectives during the Delphi process

	Initial learning objectives	Adapted learning objectives	Acceptance rate	
			First round [%]	Second round [%]
**Domain 1: Risk stratification**				
1	To be able to identify high risk situations independent of algorithm-based workflows.		85.7 (n=49)	
2	To be able to consider frailty as a parameter for risk stratification.		81.6 (n=49)	
3	~~To be able to consider patients' physical and cognitive capacity and resilience towards acute medical interventions.~~	To be able to recognise patients' physical and cognitive ability and resilience towards acute medical interventions.	59.2 (n=49)	100 (n=40)
4	To be able to correctly interpret vital signs (including respiratory rate, SpO2, pulse, blood pressure, blood sugar, mental status and pain).		87.8 (n=49)	
5	To be able to demonstrate awareness of the limitations of conventional physiological parameters in identifying older patients with serious acute illness.		83.7 (n=49)	
6	To be able to identify atypical symptoms of serious acute diseases.		79.6 (n=49)	
**Domain 2: Indicators of serious health problems**				

7	To be able to identify falls as a potential indicator of possible serious underlying health problems.		87.8 (n=49)	
8	To be able to identify a recent decline in activities of daily living as an indicator of possible serious underlying health problems.		89.8 (n=49)	
9	To be able to identify generalised weakness as an indicator of possible serious underlying health problems.		85.7 (n=49)	
10	To be able to identify altered mental status as a potential indicator of potential serious acute illness.		91.8 (n=49)	
**Domain 3: Altered mental status**				
11	To be able to identify an altered mental status in older emergency patients.		89.8 (n=49)	
12	To be able to differentiate between acute and chronic alterations in mental status.		73. 5 (n=49)	
13	~~To be able to identify common causes of acute altered mental status (e.g. infection, pain, medication, hypoxia, hypoglycaemia, urinary retention, constipation, trauma, head injury) and to initiate appropriate initial management.~~	To be able to consider common causes of acute altered mental status (e.g. pain, hypoxia, hypoglycaemia, trauma) and to initiate emergency management.	69.4 (n=49)	92.5 (n=40)
14	To be able to identify delirium as an emergency.		83.7 (n=49)	
15	To be able to recall the predisposing and precipitating factors of delirium.		75.5 (n=49)	
16		To be able to recognise diminished decision-making capacity.	65.3 (n=49)	92.5 (n=40)

17	~~To be able to address diminished decision-making capacity and deliver care that optimizes and preserves autonomy and self-determination.~~	To be able to deliver care that respects and preserves autonomy and self-determination.		95 (n=40)
**Domain 4: Clinical assessment**				
18	To be able to describe the impact of underlying cognitive disorders on the clinical assessment.		87.5 (n=48)	
19	~~To be able to recognise clinical signs of infection and sepsis and to deliver individualised management.~~	To be able to recognise clinical signs of severe infection and sepsis and to deliver initial management (e.g. intravenous fluids).	66.7 (n=48)	92.5 (n=40)
20	To be able to recognise clinical signs of cardiovascular emergencies and to deliver individualised management.		72.9 (n=48)	
21	To be able to recognise clinical signs of neurological emergencies and to deliver individualised management.		70.8 (n=48)	
22	To be able to recognise clinical signs of dehydration and malnutrition and to deliver individualised management.		85.4 (n=48)	
23	~~To be able to recognise clinical signs of mental health crises (including depression and suicidal behaviour) and to deliver individualised management.~~	To be able to recognise clinical signs of mental health crises (including depression and suicidal behaviour) and to initiate interventions (including referral to specialists).	68.8 (n=48)	95 (n=40)
24	To be able to recognise clinical signs of abuse (including physical, psychological, social abuse) and to deliver individualised management.		79.2 (n=48)	

25	~~To be able to integrate the impact of underlying gait- and movement disorders (e.g. parkinson's disease) on the clinical assessment.~~	To be able to recognise the impact of underlying gait and movement disorders (e.g. Parkinson's disease) on clinical assessment.	64. 6(n=48)	80 (n=40)
26	To be able to identify pain in patients with and without cognitive impairment (including the use of standardised assessment tools).		89.8 (n=49)	
**Domain 5: Falls**				
27	~~To be able to perform a falls assessment, including history-taking, physical examination, and functional assessment, to identify underlying causes and contributing factors.~~	To be able to perform a basic falls assessment, including history-taking, physical examination, and functional assessment.	53.1 (n=49)	75 (n=40)
28	To be able to consider influencing factors on transport decisions such as severity of injuries, clinical condition, risks and benefits of transport, patient preferences and potential underlying causes.		81.6 (n=49)	
**Domain 6: Trauma**				
29	To be able to conduct a systematic and focused evaluation of older trauma patients, considering both obvious and subtle injuries whilst assessing the severity of the trauma.		89.8 (n=49)	
30	To be able to perform individualised management for patients with low energy transfer trauma.		81.6 (n=49)	
**Domain 7: Medication**				

31	To be able to obtain a medication history (including over-the-counter products, frequency, compliance, recent changes) and to produce a structured report.		79.6 (n=49)	
32	To be able to appreciate the importance of a detailed medication list for subsequent healthcare.		89.8 (n=49)	
33	To be able to identify high risk medication (e.g. anticoagulants, anti-platelets, anti-diabetics, antiarrhythmic drugs, diuretics, cholinergic drugs).		83.7 (n=49)	
34	To be able to consider adverse drug events as possible cause of clinical presentation.		73.5 (n=49)	
35	~~To be able to adapt medication (dosages) to the needs and characteristics of geriatric patients including age-related physiological changes and comorbidities.~~	When administering drugs:<br>To be able to adapt medication (dosage) taking into consideration age-related physiological changes and comorbidities.	51 (n=49)	77.5 (n=40)
	~~To be able to apply an individually tailored treatment plan (including agent of pain medication and dosage).~~		65.3 (n=49)	
**Domain 8: Communication and medical history taking**				
36	To be able to explain the impact of person-centred communication.		85.7 (n=49)	
37	To be able to describe the role of professional communication with patients, relatives and health care providers.		89.8 (n=49)	
38	To be able to adapt communication skills to the individual patient needs to support shared decision-making.		83.7 (n=49)	

39	To be able to perform a focused medical history taking in patients with cognitive and functional impairments.		81.6 (n=49)	
40	To be able to perform a structured collateral history including social care.		79.6 (n=49)	
41	To be able to optimise preexisting sensory deficits (e.g. the use sensory aids such as visual and hearing aids) to overcome communication barriers.		87.8 (n=49)	
**Domain 9: Frailty**				
42	To be able to describe the concept of frailty and its implications.		81.6 (n=49)	
43	To be able to appreciate the risks and benefits of attending the emergency department for frail persons.		81.6 (n=49)	
44	To be able to identify frailty in older emergency patients.		75.5 (n=49)	
**Domain 10: Palliative and End-of-life care**				
45	~~To be able to identify goals based on the patient's condition, prognosis, and the potential benefits and risks of various interventions (including hospitalisation).~~	To be able to consider patient-centred healthcare goals based on the patient's condition, perspectives, and the potential benefits and risks of various interventions (e.g. hospitalisation).	53.1 (n=49)	80 (n=40)
46	~~To be able to recognise palliative care needs and to deliver physical, psychological and social support.~~	To be able to recognise palliative care needs and to initiate physical, psychological, and social support.	63.3 (n=49)	82.5 (n=40)

47	To be able to explain the importance of effective communication and compassionate support when delivering end-of-life care to patients and their families.		77.6 (n=49)	
48	~~To be able to recall the legal and regulatory frameworks surrounding end-of-life care decisions, including informed consent, surrogate decision-making, and medical futility.~~	To be able to recall the relevant legal and regulatory frameworks surrounding palliative care decisions, where available.	67.4 (n=49)	82.5 (n=40)
	~~To be able to consider advance directives and powers of attorney when provided.~~		69.4 (n=49)	
49	To be able to analyse personal attitudes, biases, and emotions related to death and dying, and develop strategies for self-care and professional resilience when providing end-of-life care.		77.6 (n=49)	
**Domain 11: Positioning and transport**				
50	To be able to consider patient-specific factors (e.g. skin problems, pain) when determining the transportation route and positioning within the ambulance.		79.6 (n=49)	
51	To be able to optimise preexisting mobility deficits (e.g. to convey patients with their mobility aids) in order to overcome barriers to mobilisation.		87.8 (n=49)	
**Domain 12: Social, psychological and legal aspects**				
52	To be able to explain negative stereotypes associated with older people.		75.5 (n=49)	

53	To be able to analyse reasons for suboptimal care, encouraging self-reflection and promoting empathy towards older individuals.		79.6 (n=49)	
54	To be able to consider the importance of different community health care/social facilities for the care of older people.		91.8 (n=49)	
55	To be able to initiate support for relatives requiring assistance.		87.8 (n=49)	
56	To be able to appreciate patients' preferences when delivering emergency care.		85.7 (n=49)	
57	To be able to integrate healthcare proxy holders (e.g. family, friends, or caregivers) when necessary.		93.9 (n=49)	
**Domain 13: Pain**		Removed, enhancing overall clarity. Corresponding learning objectives were assigned to other domains (*Domain 4: Clinical assessment, Domain 7: Medication*).		

Supplementary file 3. Detailed information on acceptance rates and adaptation of learning objectives during the Delphi process. The strike-through 'initial learning objectives' were adapted during the Delphi process and are not included in the final curriculum. n = number of ratings on the respective learning objective.
